# Supplementary figures and images for: KAT2-mediated acetylation switches the mode of PALB2 chromatin association to safeguard genome integrity
Source: eLife. 2022 Oct 21;11:e57736. doi: 10.7554/eLife.57736 (PMC9671498; doi:10.7554/eLife.57736)

**Figure 1 - source data 1B**

### Figure 1B

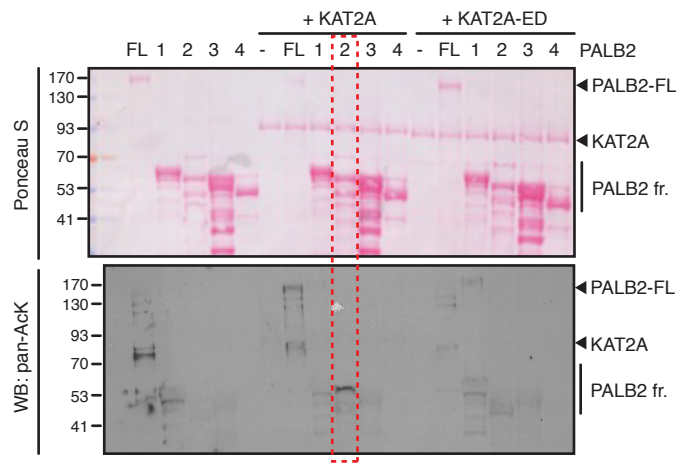

**source data**

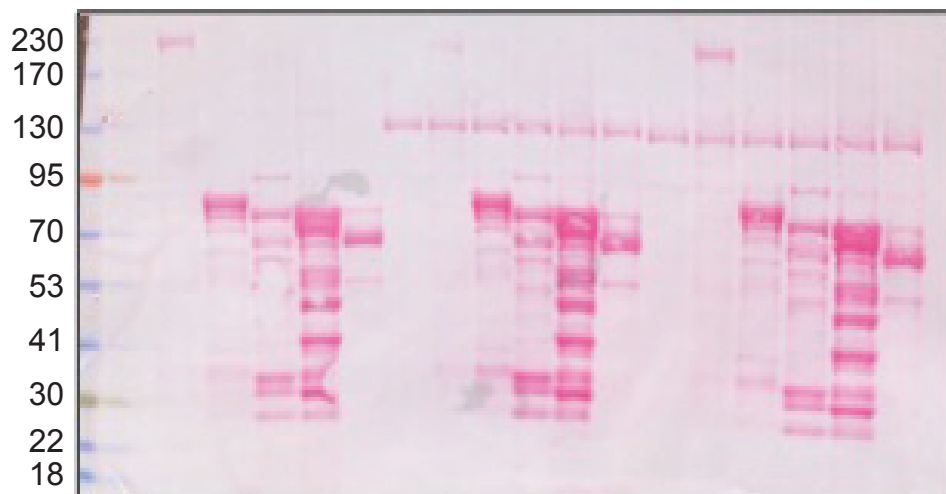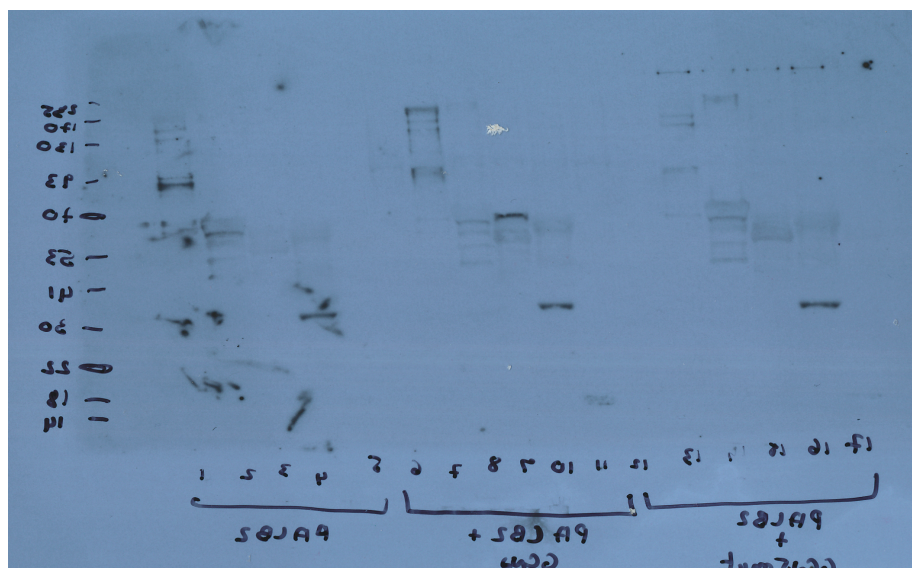

Supplement: Figure 1—source data 1. [file elife-57736-fig1-data1.pdf]

**Figure 1-figure supplement 1 - source data 1**

**Figure 1 - figure supplement 1A**

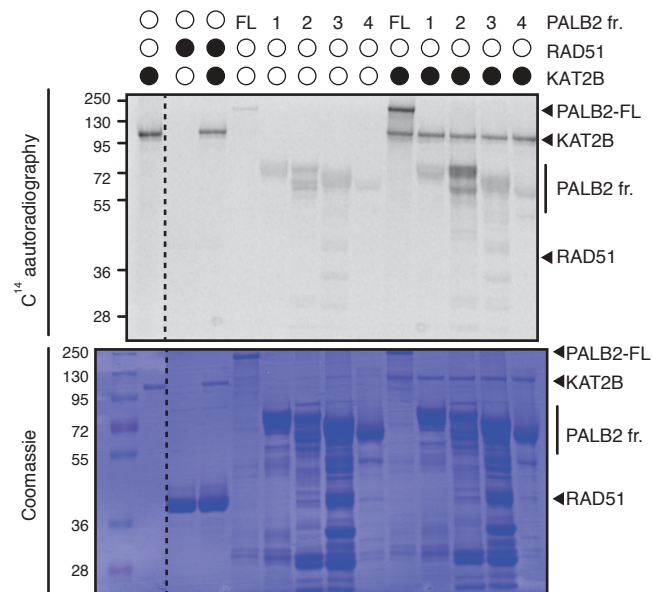

**source data**

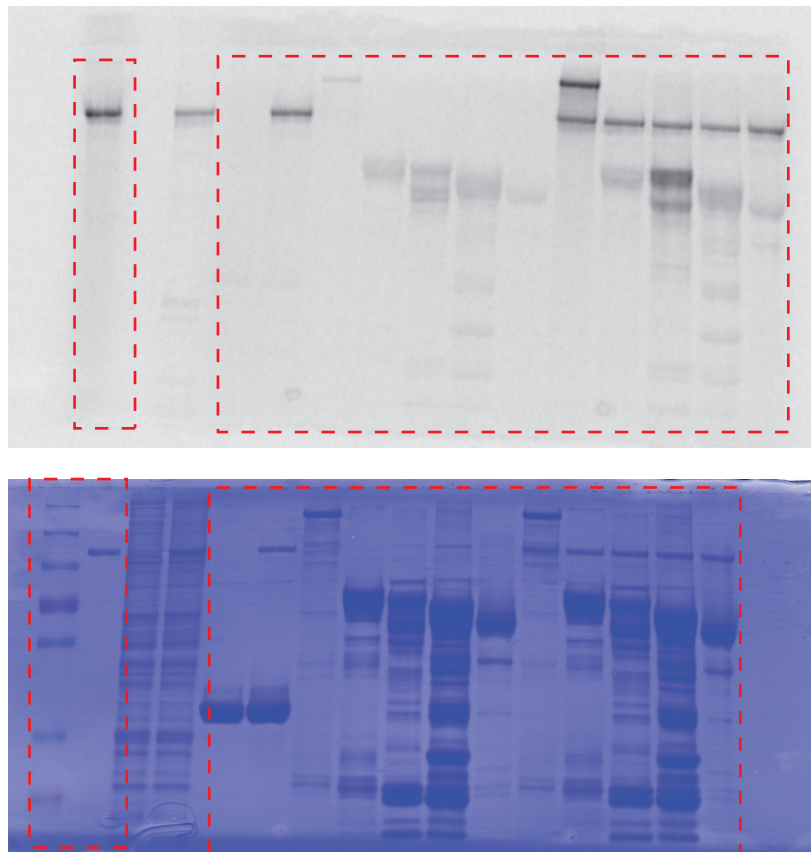

Supplement: Figure 1—figure supplement 1—source data 1. [file elife-57736-fig1-figsupp1-data1.pdf]

Figure 1-figure supplement 1 - source data 2

Figure 1 - figure supplement 1B

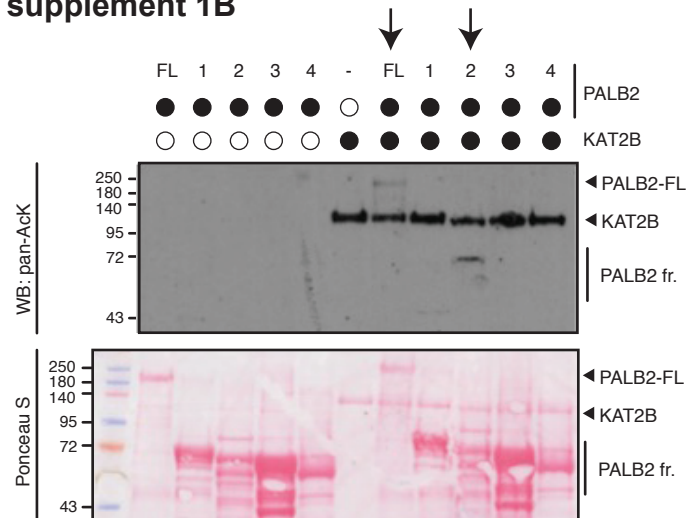

source data

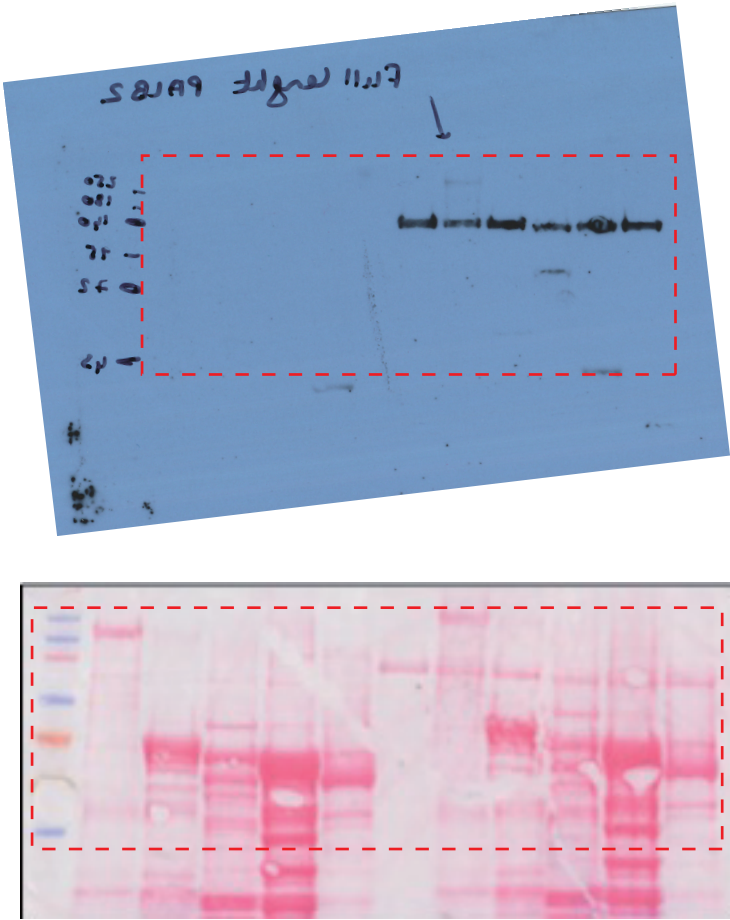

Supplement: Figure 1—figure supplement 1—source data 2. [file elife-57736-fig1-figsupp1-data2.pdf]

Figure 2B

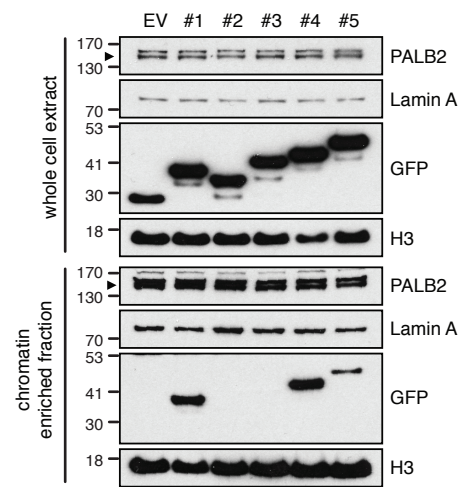

source data

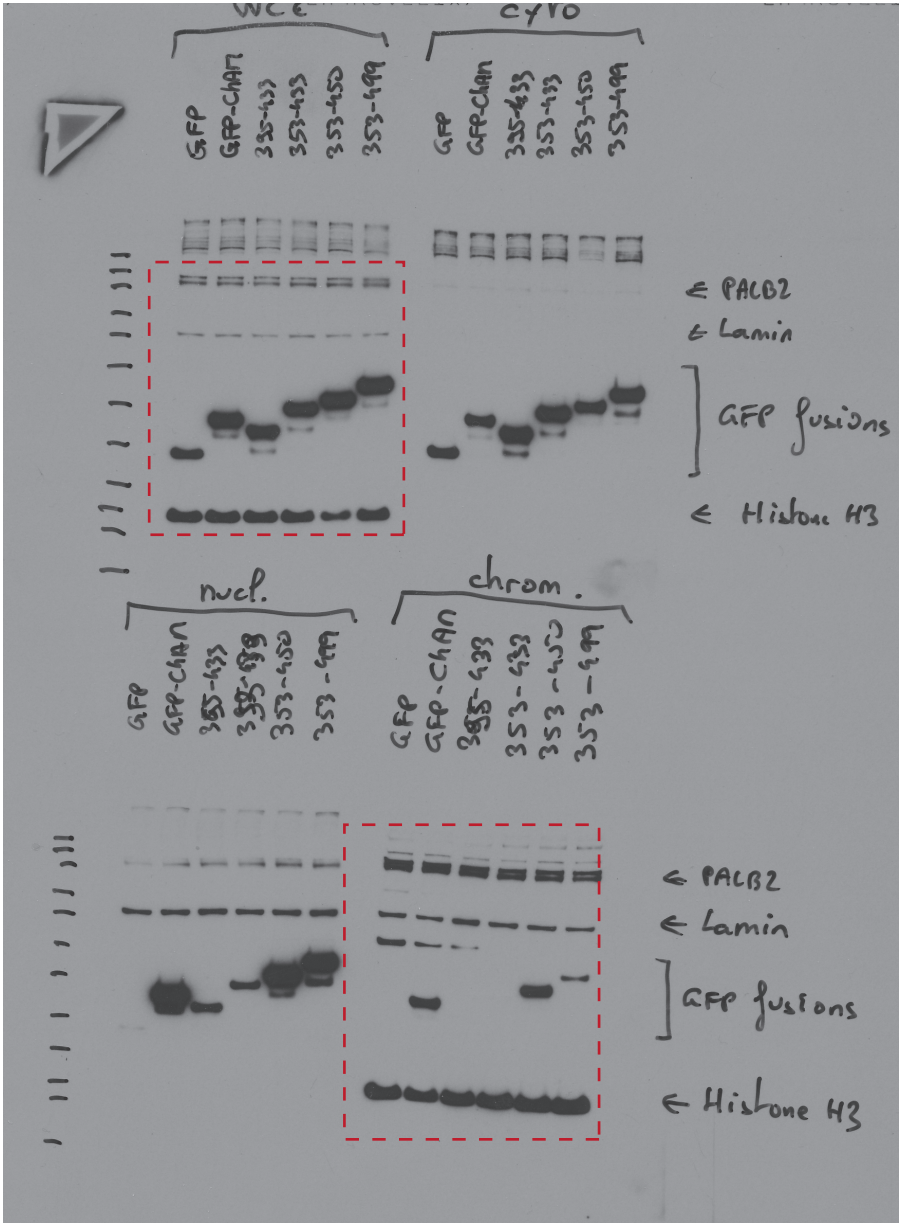

Supplement: Figure 2—source data 1. [file elife-57736-fig2-data1.pdf]

Figure 2C

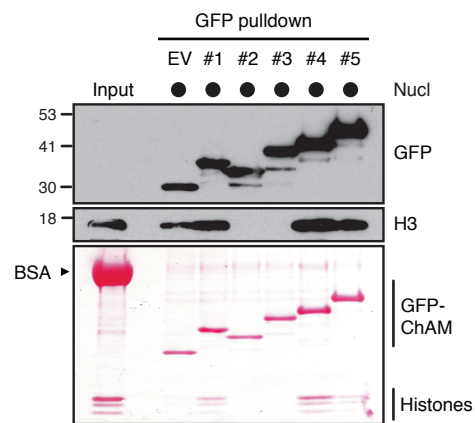

source data

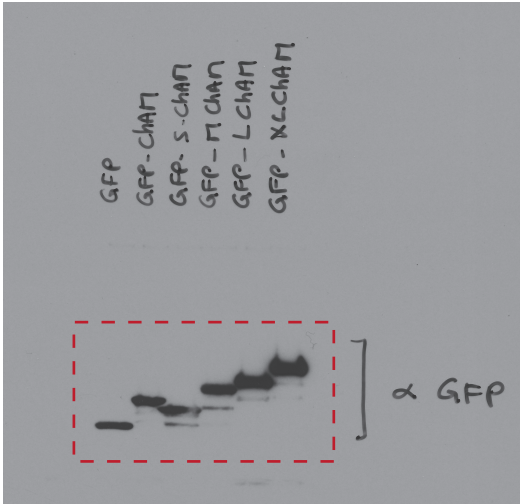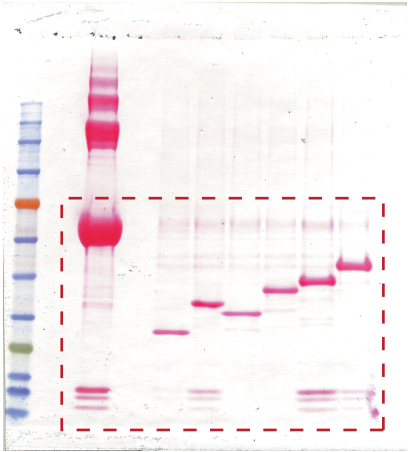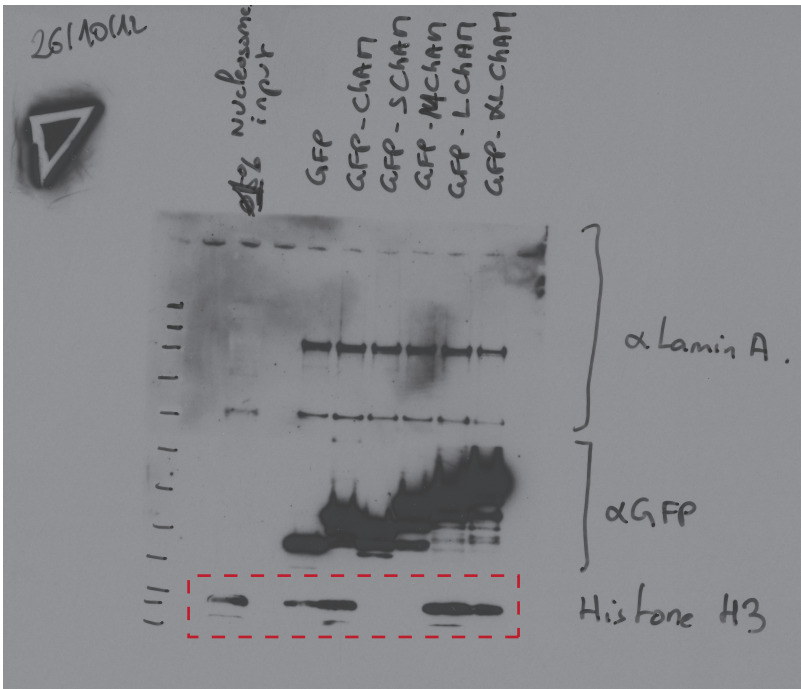

Supplement: Figure 2—source data 2. [file elife-57736-fig2-data2.pdf]

Figure 2 - source data 4

Figure 2D

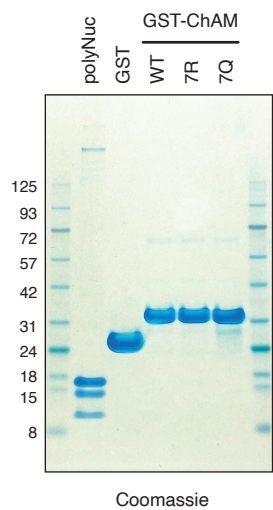

Figure 2E

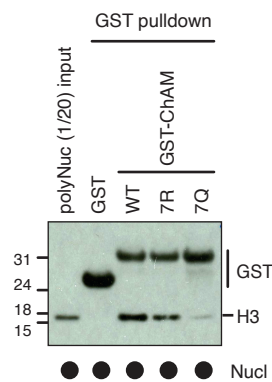

source data

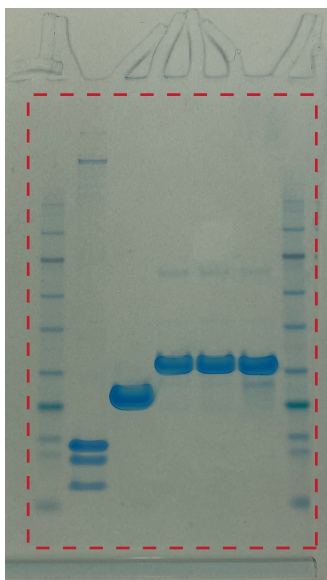

source data

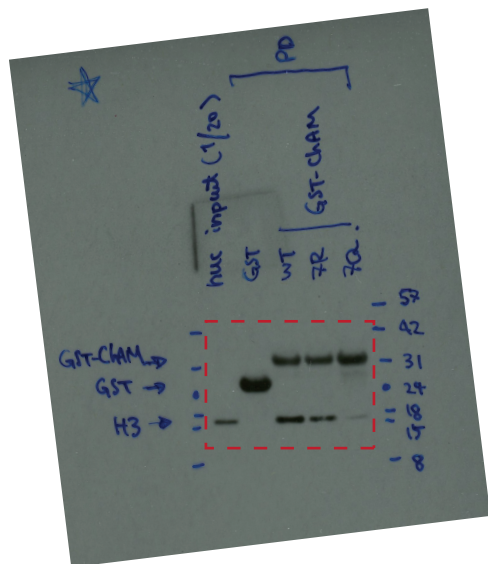

Supplement: Figure 2—source data 3. [file elife-57736-fig2-data3.pdf]

Figure 2F

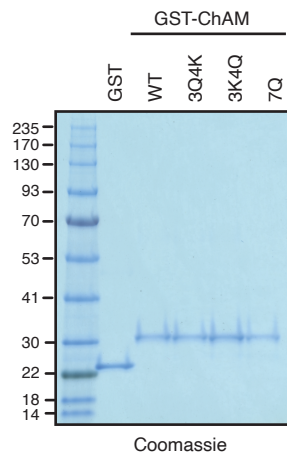

source data

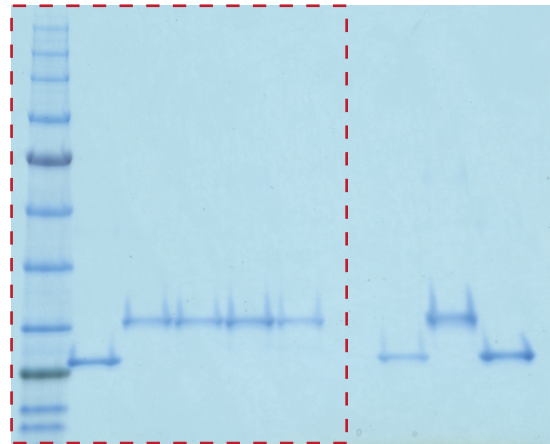

Figure 2G

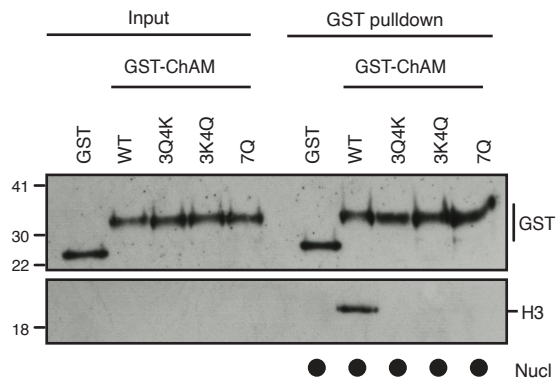

source data

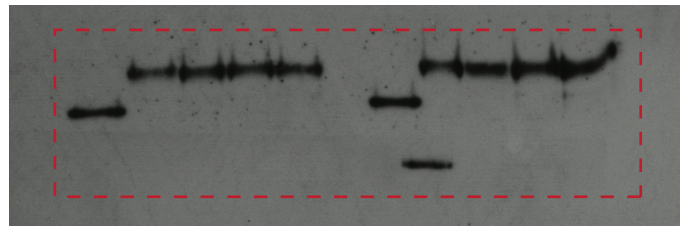

Supplement: Figure 2—source data 4. [file elife-57736-fig2-data4.pdf]

Figure 2 - figure supplement 1 - source data 1

Figure 2 - figure supplement 1B

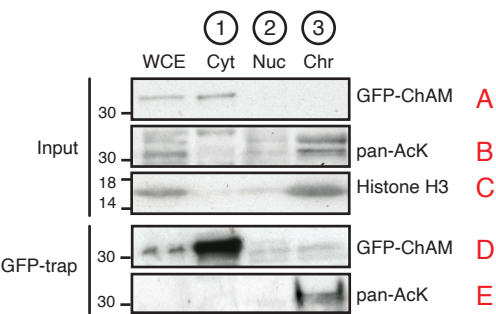

source data

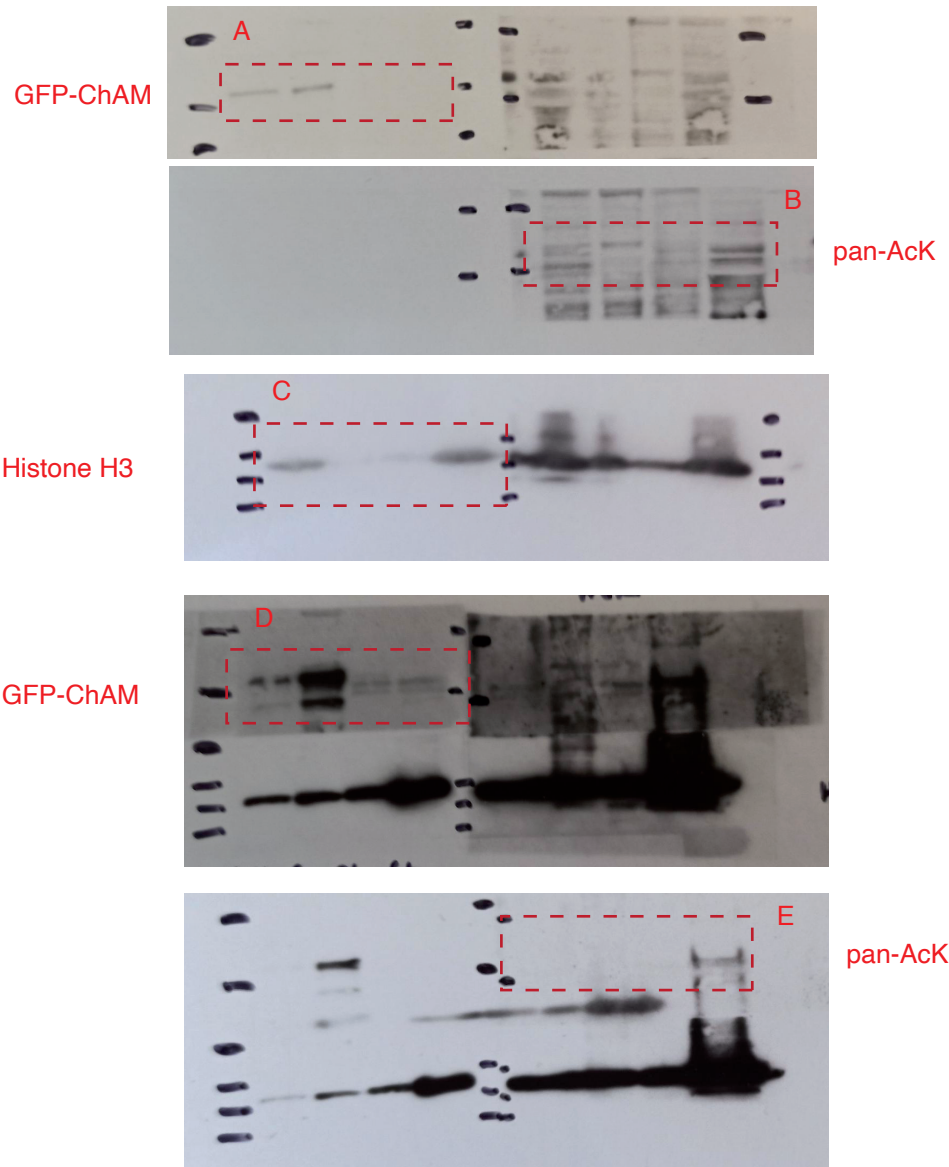

Supplement: Figure 2—figure supplement 1—source data 1. [file elife-57736-fig2-figsupp1-data1.pdf]

Figure 3B

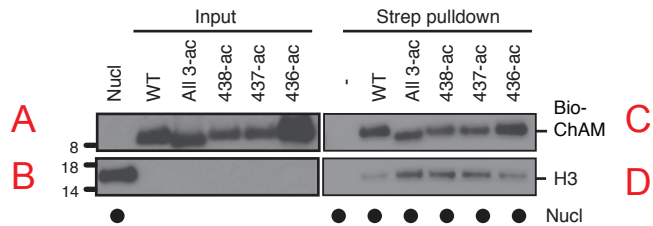

source data

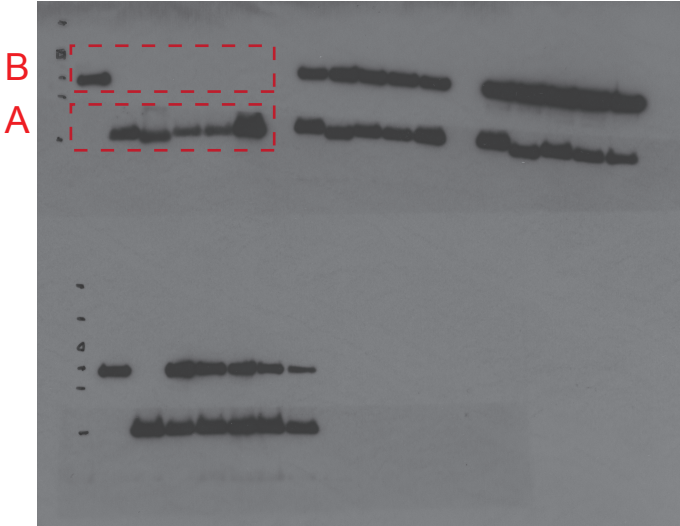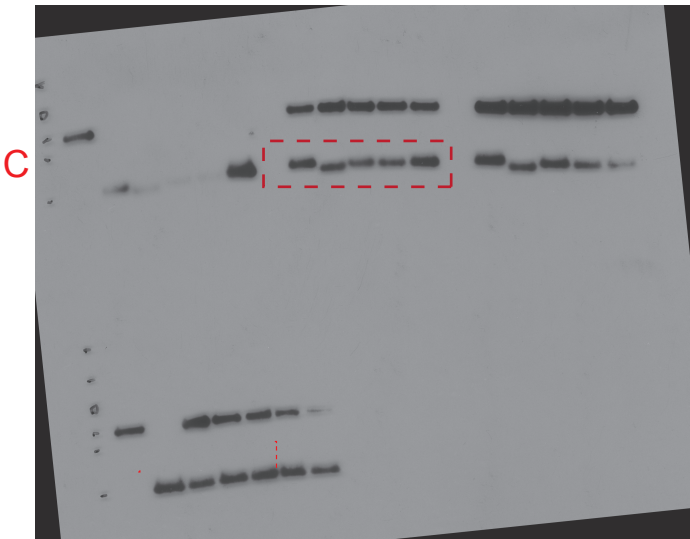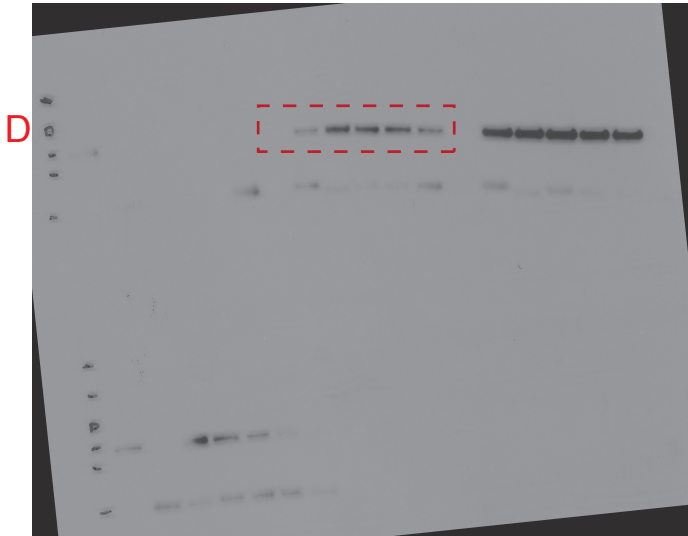

Supplement: Figure 3—source data 1. [file elife-57736-fig3-data1.pdf]

Figure 3 - figure supplement 1B

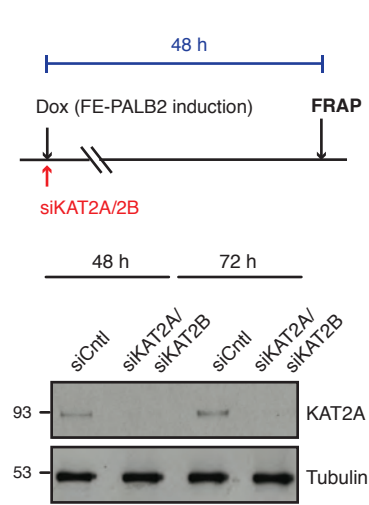

source data

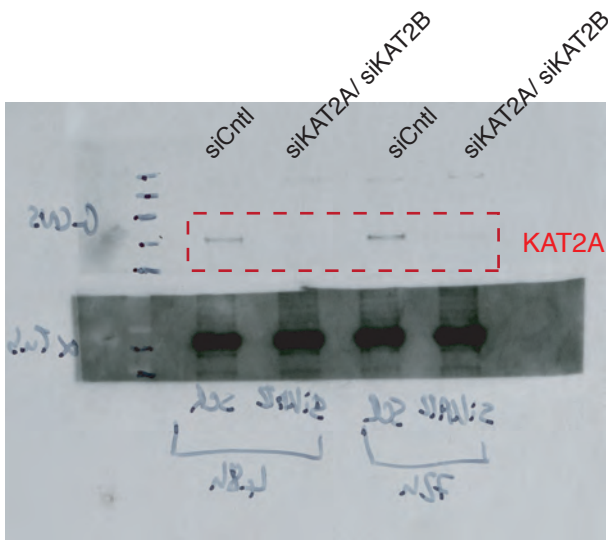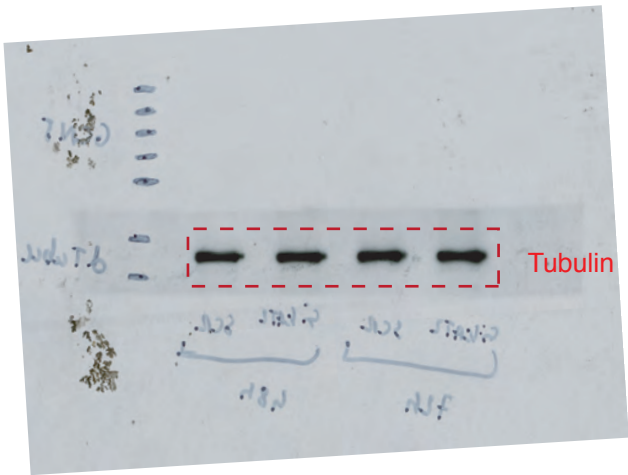

Figure 3 - figure supplement 1D

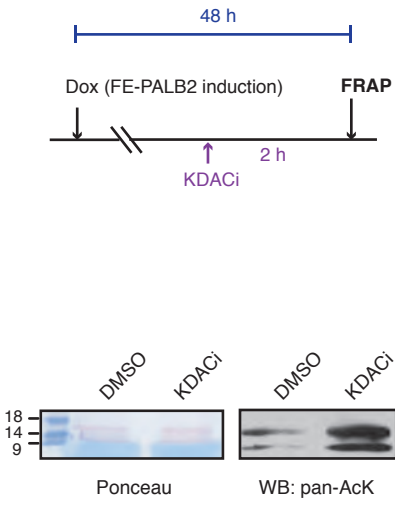

source data

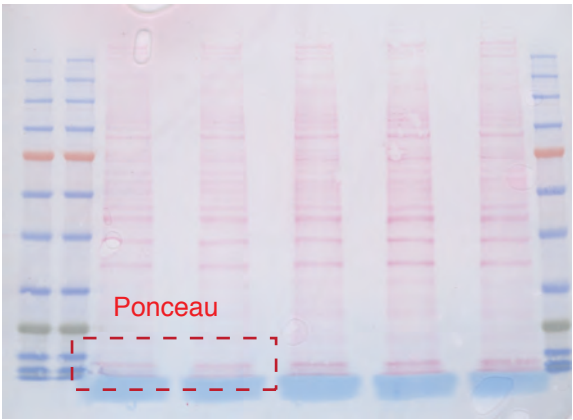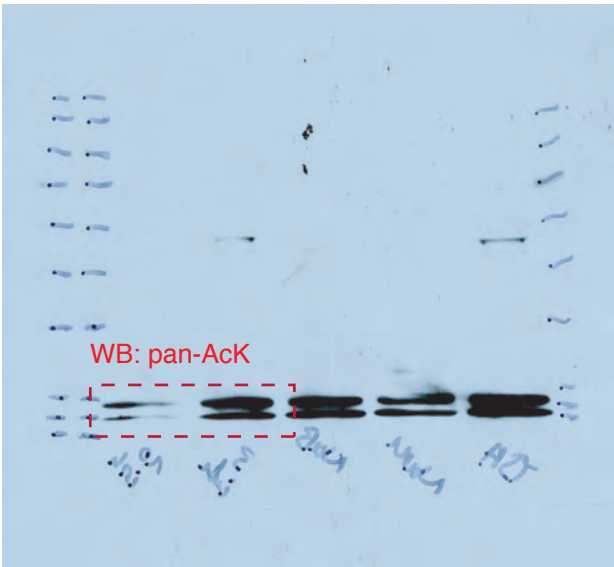

Supplement: Figure 3—figure supplement 1—source data 1. [file elife-57736-fig3-figsupp1-data1.pdf]

Figure 4B

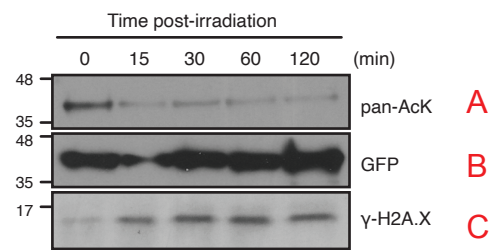

source data

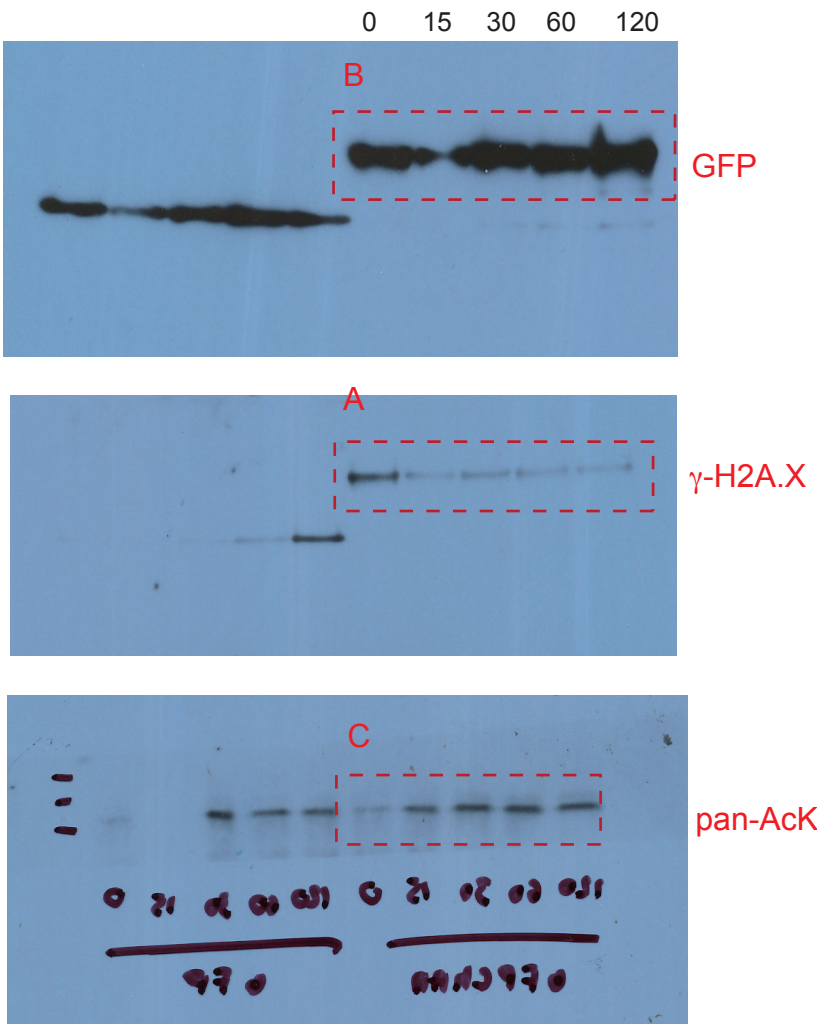

Supplement: Figure 4—source data 1. [file elife-57736-fig4-data1.pdf]

Figure 5A

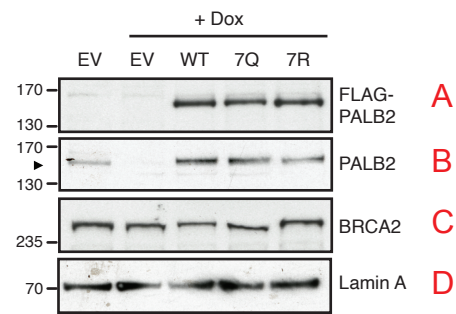

source data

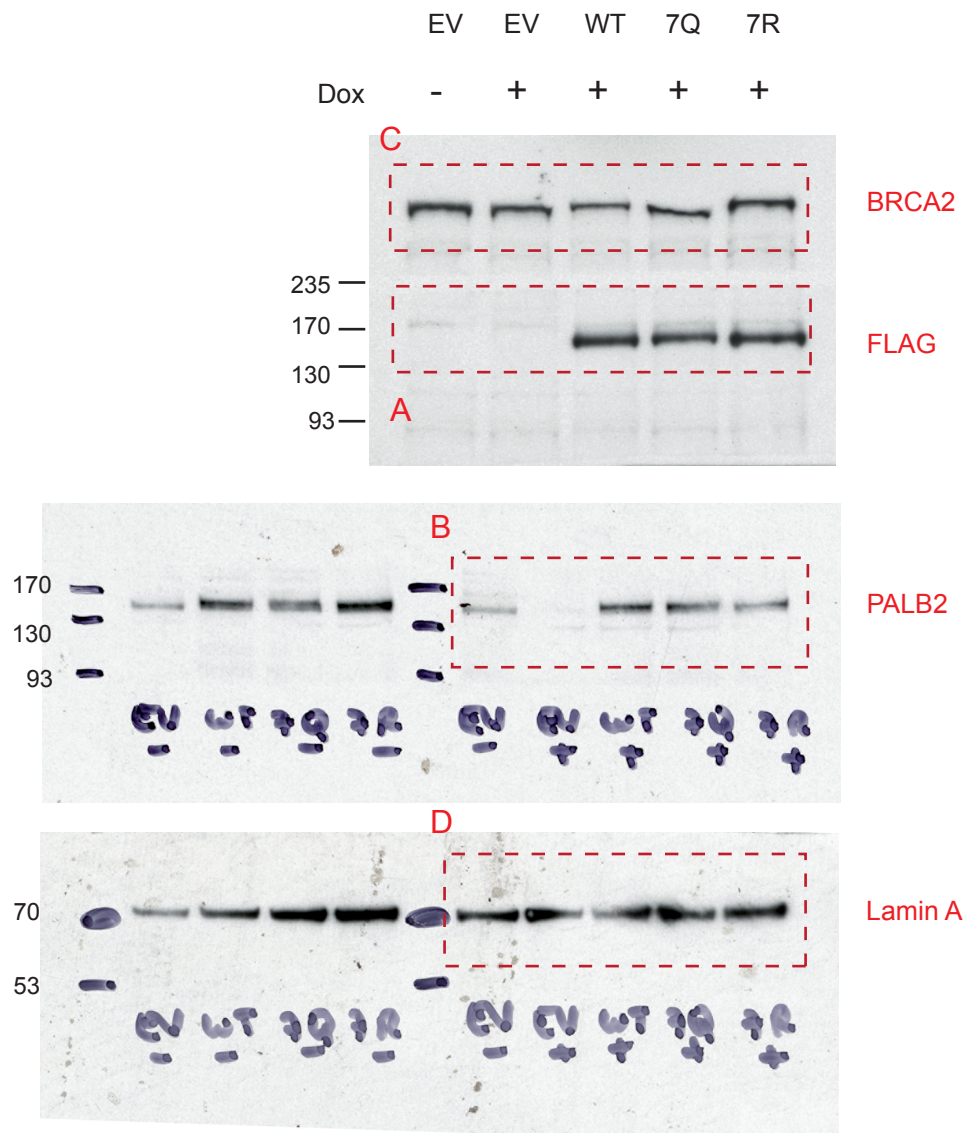

Supplement: Figure 5—source data 1. [file elife-57736-fig5-data1.pdf]

Figure 5B

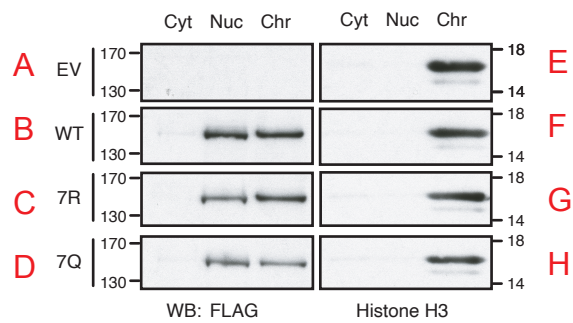

source data

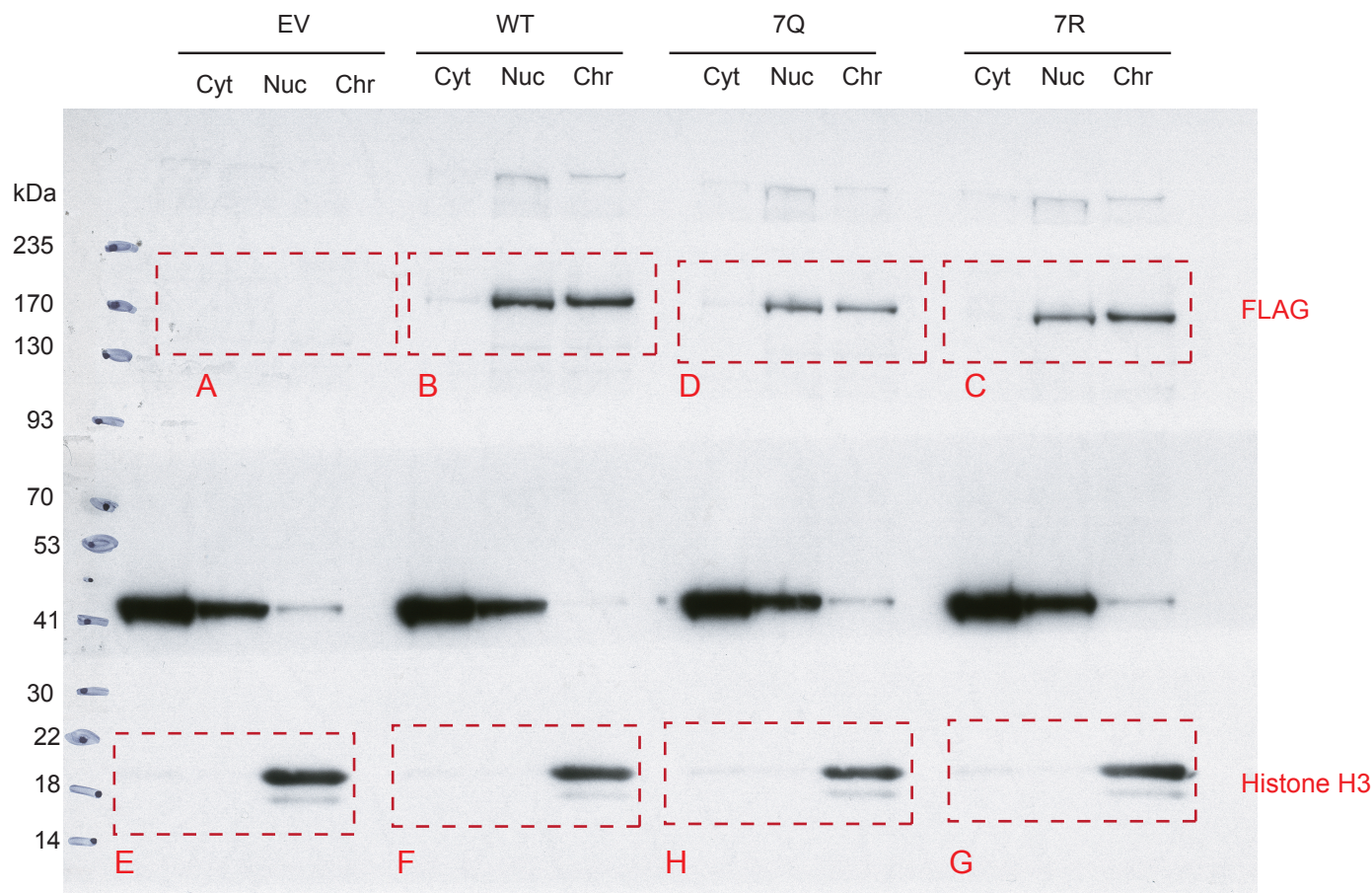

Supplement: Figure 5—source data 2. [file elife-57736-fig5-data2.pdf]

Figure 6A

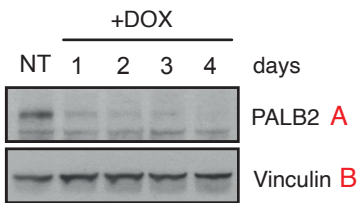

source data

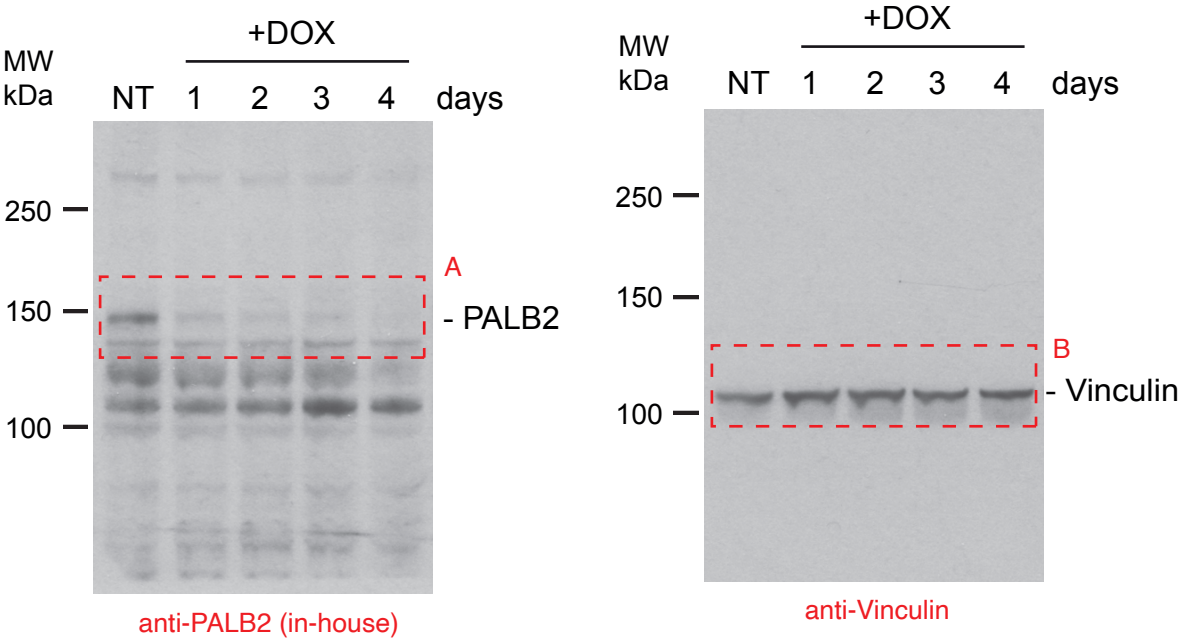

Supplement: Figure 6—source data 1. [file elife-57736-fig6-data1.pdf]

Figure 6-figure supplement 1 - right panel (FLAG-IP)

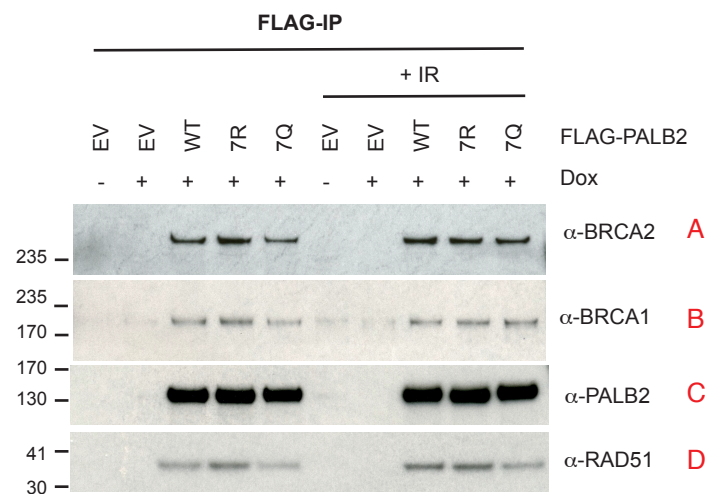

source data

Ponceau S staining

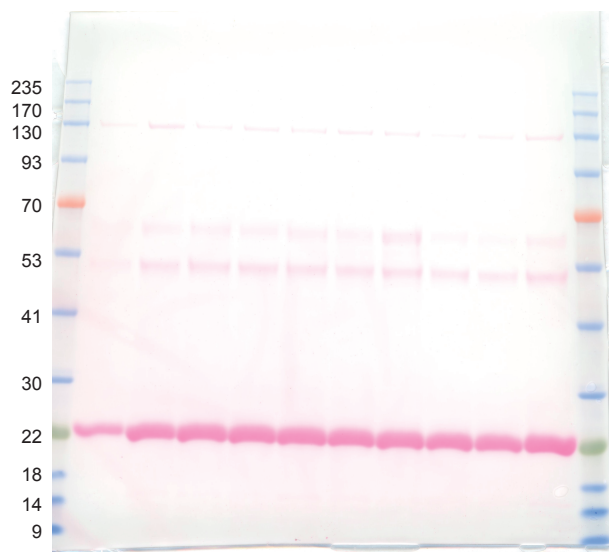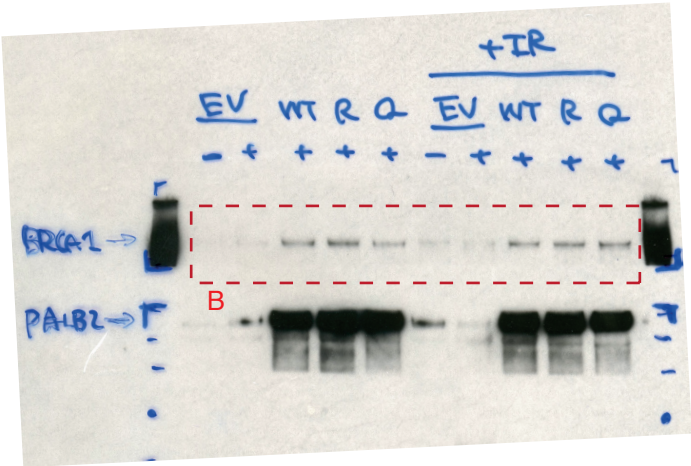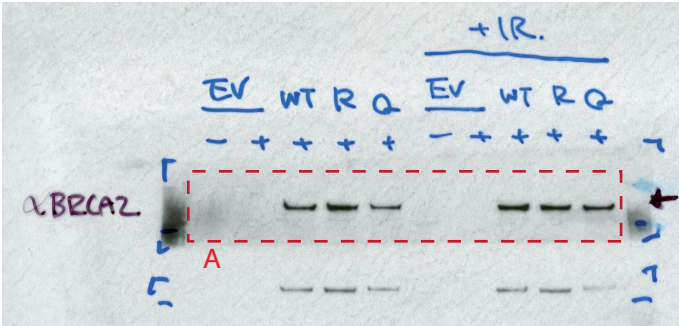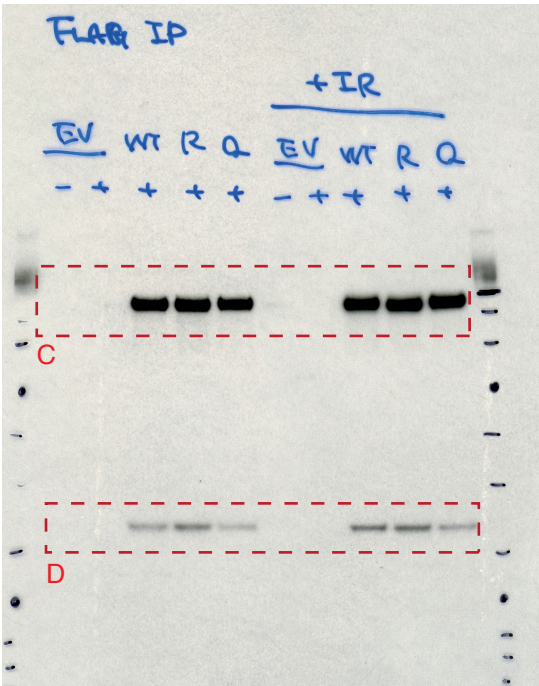

Supplement: Figure 6—figure supplement 1—source data 1. [file elife-57736-fig6-figsupp1-data1.pdf]

Figure 6-figure supplement 1 - left panel (input)

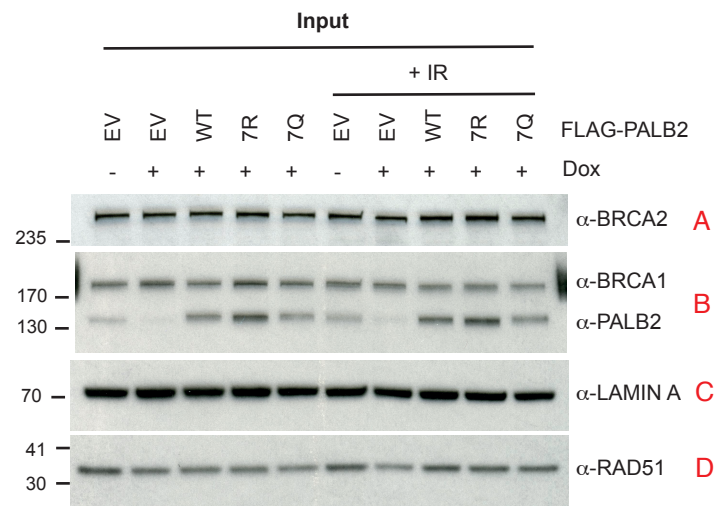

source data

Ponceau S staining

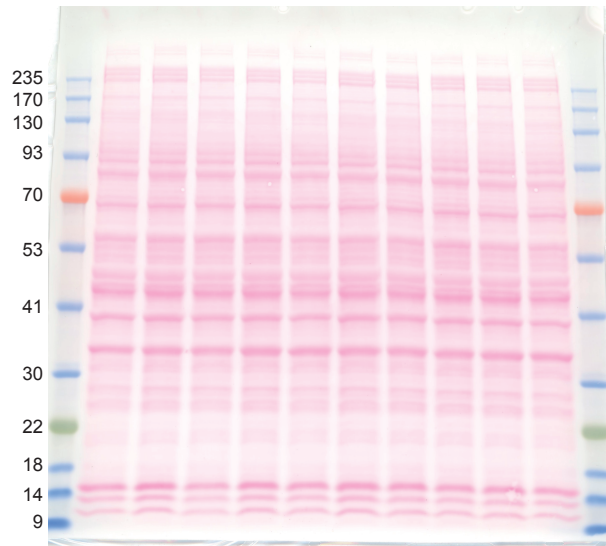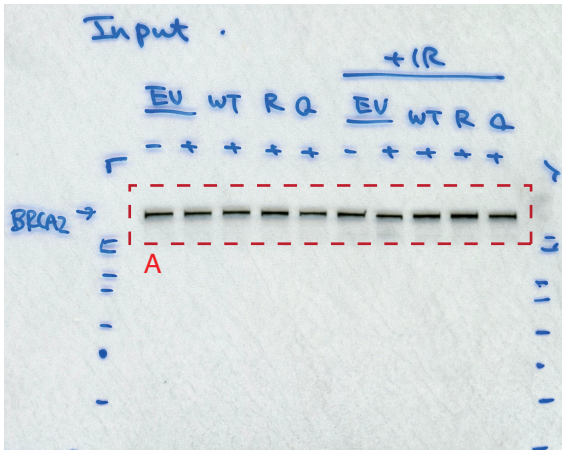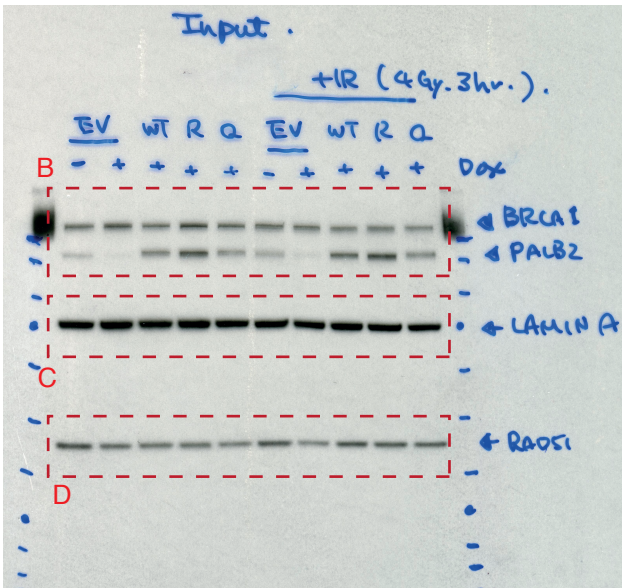

Supplement: Figure 6—figure supplement 1—source data 2. [file elife-57736-fig6-figsupp1-data2.pdf]
